# Real-Time and Accurate Drone Detection in a Video with a Static Background

**DOI:** 10.3390/s20143856

**Published:** 2020-07-10

**Authors:** Ulzhalgas Seidaliyeva, Daryn Akhmetov, Lyazzat Ilipbayeva, Eric T. Matson

**Affiliations:** 1 Department of Electrical Engineering, Telecommunications and Space Technologies, Satbayev University, Almaty 050000, Kazakhstan; useidali@purdue.edu; 2 Department of Radio Engineering, Electronics and Telecommunications, International IT university, Almaty 050000, Kazakhstan; 24168@iitu.kz (D.A.); l.ilipbayeva@edu.iitu.kz (L.I.); 3 Department of Computer and Information Technology, Purdue University, West Lafayette, IN 47907-2021, USA

**Keywords:** unmanned aerial vehicles, object detection, deep learning, computer vision, image processing, drone detection, UAV detection, visual detection

## Abstract

With the increasing number of drones, the danger of their illegal use has become relevant. This has necessitated the creation of automatic drone protection systems. One of the important tasks solved by these systems is the reliable detection of drones near guarded objects. This problem can be solved using various methods. From the point of view of the price–quality ratio, the use of video cameras for a drone detection is of great interest. However, drone detection using visual information is hampered by the large similarity of drones to other objects, such as birds or airplanes. In addition, drones can reach very high speeds, so detection should be done in real time. This paper addresses the problem of real-time drone detection with high accuracy. We divided the drone detection task into two separate tasks: the detection of moving objects and the classification of the detected object into drone, bird, and background. The moving object detection is based on background subtraction, while classification is performed using a convolutional neural network (CNN). The experimental results showed that the proposed approach can achieve an accuracy comparable to existing approaches at high processing speed. We also concluded that the main limitation of our detector is the dependence of its performance on the presence of a moving background.

## 1. Introduction

With the constant development of technology, drone companies such as DJI, Parrot, and 3DRobotics are producing different types of unmanned aerial vehicles (UAVs) or systems (UAS). Because of their accessibility and ease of use, UAVs are widely used for commercial purposes, such as the delivery of goods and medicines, surveying, the monitoring of public places, cartography, search and rescue (SAR), first aid, and agriculture. However, the wide and rapid spread of UAVs causes danger when the illegal flight of drones is used for crimes such as smuggling (the illegal transportation of goods at borders, in restricted areas, prisons, etc.), illegal video surveillance, and interference with aircraft flying. In recent years, unmanned aerial vehicles, which are publicly known as drones, have hit the headlines by flying over restricted zones and entering the high-security areas. In January 2015, a drone flown by an intoxicated government officer crashed right in front of the White House’s lawn [1]. Another accident happened in 2017 in the Canadian province of Quebec, where during landing, a plane with a light engine crashed into a UAV at an elevation of 450 m [2]. Fortunately, the plane only suffered small damage and was able to land safely. In December 2018, London’s Gatwick Airport was shut down for 36 h with reports of drones over the runway, which strangely appeared whenever the airport attempted to reopen [3]. Because of this incident, approximately 1000 flights had to be cancelled, which affected the lives of 140,000 passengers. Due to low visibility of detection, drones can be ideal tools for illegal smuggling. In April 2020, in the state of Georgia, three people were accused of arranging to transport tobacco and phones by means of a drone to a convict at Hays State Prison in Trion [4]. The given examples of drone incidents show the need to monitor the flight of drones. To guarantee security, some drone producer companies have set up no-fly zones by prohibiting drones from flying within a 25 km radius of a few sensitive zones, such as airports, prisons, power plants, and other critical facilities [5]. However, the impact of no-fly zones is exceptionally constrained, and not all drones have those built-in safeguards. Therefore, to solve this problem, the development of anti-drone systems is vigorously developing, and the problem of real-time drone detection is becoming relevant [6]. Drone detection technologies are usually divided into four categories: acoustic, visual, radio-frequency signal-based, and radar [7]. A good balance between price and detection range is achieved using visual drone detection technologies that use images of surveillance areas from cameras. One of the main disadvantages of visual drone detection is the high level of false positives caused by the visual similarity of different objects, especially when they occupy several pixels in an image [8]. As a result, a drone can be mistaken for a bird or background and vice versa. The task becomes even more difficult due to large changes in images caused by varying weather and lighting conditions. At the same time, drones can reach speeds of up to 100 miles per hour, which imposes additional requirements on the speed of detection. To address these problems, the Drone-vs-Bird detection challenge [5] was established. The challenge provides video sequences in which drones are present along with birds. The goal is to detect all drones that appear on video, while birds should not be mistaken for drones. The challenge focuses on the detection accuracy of proposed algorithms, but the execution time of the algorithms is not considered. Our objective was to develop a real-time drone detection algorithm that could achieve a competitive accuracy.

### 1.1. Drone Detection Modalities

Based on investigations conducted by academia and commercial industries, the primary modalities that can be used for drone detection and classification tasks are radar, radio frequency (RF), acoustic sensors, and camera sensors supported by computer vision algorithms [7].

#### 1.1.1. Radar-Based Drone Detection

Radar is considered to be a traditional sensor that provides the robust detection of flying objects at long-range distances and almost uninfluenced performance in unfavorable light and weather conditions [9,10]. As radar sensors are mostly designed for detecting high velocity ballistic trajectory targets such as military drones, aircrafts, and missiles, they are not suitable to detect small commercial UAVs that fly with relatively lower non-ballistic trajectory velocities [11]. While radar sensors are well-known as reliable solutions for detection, their classification abilities are not optimal [9]. Since UAVs and birds have key characteristics that often make them difficult to distinguish, the above-mentioned drawback of radar sensors makes it an unprofitable solution for the classification task of UAVs and birds. The complexity of installation and high cost of radar sensors are other reasons that necessitate a relatively low-cost anti-drone system.

#### 1.1.2. Acoustic-Based Drone Detection

Relatively low-cost acoustic detection systems use an array of acoustic sensors or microphones to classify specific acoustic patterns of UAV rotors, even in low visible environments [7]. However, the maximum operational range of these systems remains below 200–250 m. Additionally, sensitivity of these systems to environment noise, especially in urban or noisy loud areas and wind conditions, influences detection performance.

#### 1.1.3. RF-Based Drone Detection

RF-based UAV detection system is one of the most popular anti-drone systems in the market, and they detect and classify drones by their RF signatures [12]. An RF sensor is a passive listener between a UAV and its controller, and the sensor does not transfer any signal like in radar-based systems, which makes RF-based detection energy efficient. Unlike acoustic sensors, RF sensors solve the limited detection range problem by utilizing high-gain recipient antennas together with highly sensitive recipient systems to listen to UAV controller signals, and the environmental noise problem is suppressed by using some de-noising methods such as band pass filtering and wavelet decomposition [13]. However, not all drones have RF transmission, and this approach is not suitable for detecting UAVs operating autonomously without communication channels [7].

#### 1.1.4. Camera-Based Drone Detection

The detection of drones that do not have RF transmission can be performed by using low cost camera sensors based on computer vision algorithms. It is well-known that detection and classification abilities are highest when the target is visible, and camera sensors have the advantage of giving a formal vision verification that the detected object is a drone while giving extra visual information such as drone model, dimensions, and payload that other drone detection systems cannot provide [14]. A medium detection range, good localization, affordable price, and easy human interpretation are achieved using visual drone detection technologies that use images of surveillance areas from cameras. However, this modality operates poorly at nighttime and in limited visibility conditions such as in the presence of clouds, fog, and dust. To address some issues of such scenarios, thermal cameras can be utilized in combination. Thermal cameras can solve the detection issue of nighttime surveillance, and sometimes, depending on the used technology, they even can operate better in rain, snow, and fog weather conditions. However, high quality thermal cameras are used for military applications, and low cost commercial thermal cameras might fail in high humidity weather conditions or other unfavorable environmental conditions [9].

#### 1.1.5. Bi- and Multimodal Drone Detection Systems

As we can see, each of these modalities has its specific limitations, and a robust anti-drone system might be complemented by fusing several modalities. In order to develop a cost-efficient drone monitoring system, some researchers [15] considered composing a sensor network with different types of sensors. Depending on the number of sensors used for the detection task, bimodal and multimodal drone detection systems can exist [7]. To improve detection accuracy, a bimodal drone detection system can combine two different modalities such as camera array and audio assistance [16], camera and radar sensors [17], and radar and audio sensors [18]. Meanwhile, a multimodal drone detection system can be performed with the simultaneous use of acoustic arrays; optical and radar sensors [19]; or simple radar, infrared, and visible cameras—as well as an acoustic microphone array [20]. Therefore, a maximal system performance can be achieved by fusing several drone detection modalities. However, our focus is the approach that uses camera images and computer vision algorithms.

### 1.2. Related Work

#### 1.2.1. UAV Detection and Classification

Drone detection based on visual data (image or video) can be performed using handcrafted feature-based methods [8,21,22] and deep learning-based [6,23,24,25] algorithms. Handcrafted feature-based methods are based on traditional machine learning algorithms by using traditional descriptors such as scale-invariant feature transform (SIFT), histogram of oriented gradients (HOG), Haar, local binary pattern (LBP), deformable parts model (DPM), and generic Fourier descriptor (GFD) that provide low-level handcrafted features (edges, drops, blobs, and color information) and classical classifiers (support vector machine (SVM), AdaBoost)), whereas the second category relies on the learned features using two-stage (region-based convolutional neural network (R-CNN), Fast R-CNN, Faster R-CNN, and Mask R-CNN) and single-stage (single shot detector (SSD), RetinaNet, and you only look once (YOLO)) deep object detectors.

Drone detection using handcrafted feature-based methods: Unlu et al. [21] developed GFD vision-based features that are invariant to translation and rotation changes to describe the binary forms (such as silhouettes) of drones and birds. In accordance with the system proposed by the authors, the silhouette of a moving object is obtained using a fixed wide-angle camera and a background subtraction algorithm—the region growing algorithm—is used to separate the pixels of the object from the background. In order to avoid the loss of any form information morphological operations are not used after the image segmentation phase, GFD is calculated after the normalization and centering of the silhouette; finally, GFD signals are classified into birds and drones through a neural network consisting approximately 10,000 neurons. To teach their system, the authors created a dataset including 410 drone images and 930 bird images collected from open sources. Training and testing on the custom dataset were performed by using five-fold cross validation. In the test data, CNN classification accuracy was 85.08%, whereas the proposed GFD method showed an accuracy of 93.10%, and the CNN architecture significantly increased the classification efficiency of a small dataset by including the GFD signal vector before classifying the neural network. In [8], the authors proposed two methods of detecting and tracking an unmanned aerial vehicles at a distance of no more than 350 feet during the daytime using image processing and motion detection to control movement and to extract the drone detected by machine learning. The authors made a comparative analysis of the MATLAB, OpenCV, and EmguCV packages currently used in image processing and object detection, and they used OpenCV in their work. According to the proposed system, to reduce the memory, the RGB image captured by a USB camera is converted to grayscale, and the adaptive threshold method is used to adjust the noise level of the image depending on the light condition by setting the threshold value to 60. To eliminate some noise, a dilation morphological operation is used by enlarging the image until it is clearly visible, including a blob tracking algorithm to hold the object and the Dlib technique if the object moves in three frames. To test the proposed system, the authors tested four types of drones (Phantom 4 Pro, Agras MG-1s, Pocket Drone JY019, and Mavic Pro), as well as other objects (birds and balloons). Wang et al. [22] proposed a simple, fast, and efficient detection system for unmanned aerial vehicles based on video images shot with static cameras that covers a large area and is very economical. The method of temporal median background subtraction was used to identify moving objects in a static video camera, and then global Fourier descriptors and local HOG features were obtained from images of moving objects. As a result, the combined Fourier descriptor (FD) and HOG features were sent to the SVM classifier, which performed classification and recognition. To prepare a dataset, the authors converted 10 videos of the unmanned quadcopter Dajiang Phantom 4, taken in various positions, into a series of images; as a result, the drones became a positive class, and other objects such as leaves and buildings were manually annotated as a negative class. For the recognition of “drone” and “non-drone” objects, FD, HOG, and the proposed FD and HOG algorithms were used, the overall accuracy of the proposed recognition method was 98%. The authors also experimentally proved that the proposed FD and HOG algorithm, even with a small dataset, could perform the task of classifying birds and drones with a greater accuracy than the GFD algorithm.

Drone detection using deep learning-based methods: Manja Wu et al. [6] developed a real-time drone detector using the deep learning method. Since training a reliable detector requires a large number of training images, the authors first developed a semi-automatic dataset with a KCF (kernelized correlation filter) tracker instead of manual labeling. The semi-automatic method of labeling datasets based on the KCF tracker accelerated the process of preprocessing the trained images. The authors developed the YOLOv2 deep learning model by changing the resolution structure of input images and adjusting the size parameters of the anchor box. To get the detection network, the authors removed the last convolution layer of Darknet19, which was previously trained on ImageNet dataset, and added three 3 × 3 convolution layers with 1024 filters and one 1 × 1 convolution layer with 30 filters at the end of the network. The network was trained using a public-domain USC (University of Southern California) drone dataset and an anti-drone dataset labeled with a KCF tracker. The 2 and 4 GB graphics processing unit (GPU)-random-access memory (RAM) configurations were used to test the detector’s operation in real time and at a low cost. With 2 GB of GPU-RAM, the processing speed reached 19 frames per second (FPS), whereas with 4 GB of GPU-RAM, the processing speed reached 33 FPS. Through various experiments, the authors achieved good results in real-time detection using the proposed detector at an affordable price for the system. Yoshihashi et al. [23] proposed a new integrated detection and control system using information on the movement of small flying objects. This system, called the recurrent convolutional network (RCN), consists of four modules, each of which performs a specific task: a convolutional layer, convolutional long short-term memory (ConvLSTM), a cross-correlation layer, and a fully connected layer. The authors used training methods for AlexNet and Visual Geometry Group-16 (VGG-16) tuning systems without training the system from scratch. To evaluate the system, it was first tested on a bird dataset to detect birds around a wind farm, and then the system was tested on a drone dataset of 20 manually captured video components to check whether the system could be applied to other flying objects. The experimental results presented in the form of receiver operating characteristics (ROC) curves showed that the proposed system gave better results than previous solutions. In [24], the authors proposed a drone tracking system that provides the exact location of the drone in the input image based on deep learning. The proposed system consists of detection and tracking modules that complement each other to achieve high performance. While the Faster R-CNN drone detection module detects and localizes the drone from static images, an object tracking module called MDNet (multi-domain network) determines the position of the drone in the next frame based on its position in the current frame, which allows one to identify a drone only in a certain area without looking for the entire frame. To prepare the dataset, the authors used a publicly available drone dataset consisting of 30 YouTube videos shot using different drone models and a USC drone dataset consisting of 30 videos shot using one drone model. Since the number of static drone images for the drone tracking problem was very limited and labeling is a laborious task, the authors developed a method for increasing data based on a model that generates training images and automatically annotates the position of the drone in each frame. The main idea of this technique is to cut out drone images in the background and place them on top of background images. The experiment results showed that, despite training in synthetic data, the proposed system worked well on realistic images of drones against a complex background. Peng et al. [25] used the physical rendering instrumentation tool (PBRT) to solve the problem of limited visual data by creating photorealistic images of UAVs. The authors developed a large-scale training set of 60,480 rendered images, choosing different positions and orientations of UAVs, 3D models, external materials, internal and external camera characteristics, environmental maps, and the post-processing of rendered images. To detect unmanned aerial vehicles, the Faster R-CNN network was precisely tuned to Detectron, recommended by Facebook artificial intelligence (AI) research, using the weights of the basic ResNet-101 model. On the basis of experimental results, the Faster R-CNN network, trained on rendered images, showed an average accuracy of 80.69% in the manually annotated UAV test set, 43.03% in the pre-trained COCO (Common Objects in Context) 2014 dataset, and 43.36% in the PASCAL VOC (Visual Object Classes) 2012 dataset, and it showed an average precision of 56.28% in the rendered training set. According to the results of the experiment, the average precision (AP) of the Faster R-CNN detection network trained on rendered images was relatively higher compared to other methods.

Hu et al. [26] adapted and fine-tuned the YOLOv3 detector [27] to detect drones. The authors collected a dataset consisting of images of drones on which they trained the detector. The video processing speed reached 56.3 frames per second.

The best results in drone detection have been achieved by detectors based on deep learning. This is evidenced by the fact that most studies on the detection of drones [28,29,30,31] have partially or totally relied on CNNs for solving the problem.

#### 1.2.2. Drone-vs.-Bird Challenge

The primary related works on UAV detection are the methods proposed specifically for the Drone-vs-Bird detection challenge [5], which was organized in 2017 and 2019. The main goal of the challenge is to detect and distinguish drones from birds in short videos taken from a large distance by a static camera. To perform flying object detection, Saqib et al. [32] evaluated the Faster R-CNN [33] object detector with different CNN backbones. According to the results of the conducted experiment, the Faster R-CNN with VGG-16 backbone network performed better than other networks by reaching 0.66 mAP. The authors concluded that the experiment results might be improved by annotating birds as a separate class, which could reduce false positive factors and enable the trained model to accurately distinguish birds and drones. C. Aker et al. [34] solved the problem of predicting the location of a drone in video and distinguishing drones from birds by adapting and finetuning single-stage YOLOv2 [35] algorithm. An artificial dataset was created by mixing real images of drones and birds subtracted from their backgrounds with frames of coastal area videos. The proposed network was evaluated by using precision–recall (PR) curves, where precision and recall values reached the value of 0.9 at the same time. Nalamati et al. [28] examined the problem of detecting small drones using state-of-the-art deep learning methods such as the Faster R-CNN [33] and SSD [36]. Inception v2 [37] and ResNet-101 [31] were chosen as the backbone networks. The authors fine-tuned backbone networks using the Drone-vs-Bird challenge dataset, which consisted of 8771 frames extracted from 11 Moving Picture Experts Group (MPEG4)-coded videos. For each algorithm, two cases were considered when the drone is close to the camera, i.e., when it is big, and when the drone is far from the camera, that is small. According to the results of the conducted experiment, in the first case, all the algorithms were able to detect a drone, and in the second case, the Faster R-CNN with ResNet-101 backbone network could successfully detect both drones when two drones appeared in the frame simultaneously at large distances, whereas the Faster R-CNN with the Inception v2 backbone network was only able to detect one of two drones. The single-stage SSD detector, on the other hand, could not detect both long-range drones and was ineffective at detecting small objects. According to the conducted evaluation and the challenge results, the Faster R-CNN with ResNet-101 performed best and achieved recall and precision values of 0.15 and 0.1, respectively. The authors did not pay attention to the detection time, but in future work, they will use the detection time as a key indicator to evaluate the effectiveness of the proposed model in real-time drone detection application. David de la Iglesia et al. [38] proposed an approach based on the RetinaNet [39] object detector. To perform drone predictions at different scales, the feature pyramid network (FPN) [40] architecture was used, where lower pyramidal levels are responsible for detecting small objects and upper levels are focused on larger objects. The ResNet-50-D [29] was used as a backbone network that was trained on the Drone-vs-Bird and Purdue UAV [41] datasets. The precision attained on the Drone-vs-Bird challenge was around 0.52, while recall was 0.34. In addition, experimental results in this work showed that the use of motion information can significantly increase the accuracy of detection. According to the challenge results, the F1 score of this approach reached 0.41. In order to improve the accuracy of detection of existing detectors, some approaches used the additional processing of the data. For example, Magoulianitis et al. [30] pre-processed images with deep CNN with skip connection and network in network (DCSCN) super-resolution technique [42] before using the Faster-RCNN detector. As a result, the detector became capable of detecting very distant drones, increasing its recall performance. The results obtained on challenge were 0.59 for recall and 0.79 for precision. In some works [14,43], the solution was divided into two stages. In the first stage, all objects that are highly likely drones are detected. In the second stage, a high-precision classifier is applied to the detections to reduce the number of false positives. For example, Schumann et al. [43] designed a flying object detector using the median background subtraction or deep neural network-based region proposal network (RPN) algorithm. After that, the detected flying objects were classified into drones, birds, and clutter by using the proposed CNN classifier optimized for small targets. In order to train a robust classifier, the authors created their own dataset containing totally 10,386 images of drones, birds, and backgrounds. The proposed framework was evaluated by using five static camera sequences and one moving camera sequence of the Drone-vs-Bird challenge dataset. All appearing birds in the video sequences were manually annotated. The classification of flying objects for different input image sizes such as 16 × 16, 32 × 32, and 64 × 64 was performed separately for the author’s own dataset and the Drone-vs-Bird challenge dataset. To participate in the 2017 Drone-vs.-Bird challenge, the authors proposed a VGG-conv5 RPN detector optimized for 16 × 16 image size, and, based on the challenge metric results, this team took first place in that competition. Celine Craye et al. [14] developed two separate networks—the semantic segmentation network U-net [44] for the detection stage and ResNetV2 network [45] for the classification stage. By achieving a recall value of 0.71 and a precision value of 0.76, their approach won the Drone-vs-Bird detection challenge in 2019.

## 2. Proposed Approach

**Figure 1:**
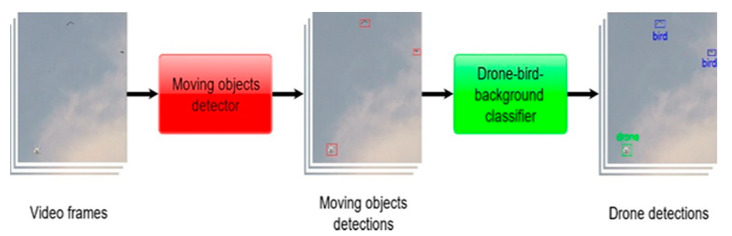
The proposed drone detection pipeline.

In this work, we focused on the real-time detection of drones in the scene with a static background. As illustrated in Figure 1, our approach consists of 2 modules: a moving object detector and a drone–bird-background classifier.

The motion detector was based on a background subtraction method. The outputs of this module are all moving objects in the scene. All the detections are fed into a classifier, which differentiates drones from other moving objects. The classifier is a CNN that was trained on the dataset of images of birds, drones, and backgrounds we collected.

### 2.1. Background Subtraction Method

There are several methods that are used for detecting flying or moving objects from a video sequence such as background subtraction, optical flow method, edge detection, and frame differencing [45]. Optical flow is used for motion estimation in a video and detects moving objects on the basis of objects’ relative velocities in the scene. The complicated calculation of the optical flow method makes it inapplicable for real-time detection tasks [45]. By calculating the difference between the current and the previous frames of a video sequence, a frame differencing algorithm extracts the moving objects. Despite its advantages, including quick implementation, flexibility to dynamic changes of the scene, and relatively low computation, frame differencing is generally inefficient for extracting all the relevant pixels of the moving regions [45]. To detect a foreground object from the background of a video sequence background, the subtraction method is used. The background subtraction method is considered one of the widely used detection methods because of its fast and accurate detection, which makes it applicable for real-time detection. Additionally, it is easy to implement. The main drawback of this method is its invalidity for moving cameras, because each frame has different background. In our case, we focused on a video with a static background, and all the short videos of the Drone-vs-Bird detection challenge dataset were taken from a large distance by a static camera. Therefore, our motion detector was based on a background subtraction method.

#### Moving Objects Detection

**Figure 2:**
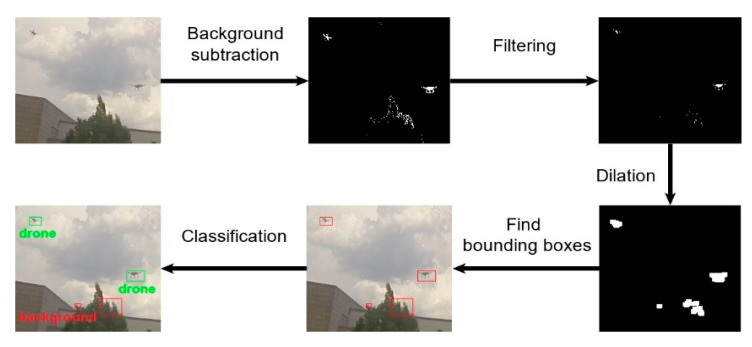
All steps of the proposed drone detection algorithm.

The task of a motion detector is to detect all objects moving in a scene. The performance of this module was evaluated by its recall value. We conducted experimental studies of various motion detectors using the Drone-vs-Bird challenge dataset. The greatest recall was achieved by the motion detector based on the two-points background subtraction algorithm [46]. The output of the common background subtraction algorithm is a binary image in which the pixels that change their values in the next frame take the value of 1. The unchanged pixels are set to zero. In addition to moving objects, the output image contains noise in the form of single pixels distributed throughout the image. To remove this noise, the output binary image is filtered. An example of a filtering result is shown in Figure 2.

Next, dilation is performed to connect closely spaced pixels. This operation reduces the number of individual regions that are checked by the classifier, therefore increasing the processing speed of the detector. The last step of the moving object detector is to find the bounding boxes covering the regions found in the previous step. All found bounding boxes are sent to the drone–bird-background classifier.

### 2.2. CNN Image Classification

Audio, image, and text classification tasks are mostly performed using artificial neural networks. For image classification, CNNs are mainly used [45]. Usually, a CNN consists of three primary layers: the convolution, pooling, and fully connected (FC) layers. Convolution layers are the main building blocks of CNN models. Convolution is a mathematical operation for combining two sets of information. Convolution layers consist of filters and feature maps. A convolution operation is performed by sliding the filter along the input. In each place, the element-wise multiplication of the matrices is performed, and the result is summed. This sum is fed into the feature map. That is, trained filters are used to extract important features of the input, and the feature map is the output of the filter applied to the previous layer. The size of the filter that performs the convolution operation is always an odd number size (1 × 1, 3 × 3, 5 × 5, 7 × 7, 11 × 11, etc.). Deep learning is commonly used to solve non-linear problems. The values obtained from the product of matrices in the convolutional layer are linear. To convert values to non-linear, after each convolutional layer, a non-linear activation function (elu, selu, relu, tanh, sigmoid, etc.) is usually used. The pooling layer is periodically added into a CNN’s architecture. Its main function is to reduce the image size and compress each feature map by taking maximum pixel values in the grid. Most CNN architectures use max pooling. Max pooling uses a 2 × 2 window with stride of 2 and takes the largest elements of the input feature map; as a result, the output of the feature map is half the size. After going through the processes described above, the model is able to understand the features. The fully connected layer comes after the convolution, activation, and pooling layers. The outputs of convolution and pooling layers are always three-dimensional (3D), but a FC layer expects a one-dimensional vector of numbers. Therefore, we flatten the output of a pooling layer to a vector, and it becomes the input of a FC layer [47]. Then, it is inserted into the nodes of the neural network, which performs the classification. Different CNN architectures exist, such as LeNet, AlexNet, VGGNet (VGG-16 and VGG-19), GoogLeNet (Inception v1), ResNet (ResNet-18, ResNet-34, ResNet-50, etc.), and MobileNet (MobileNetV1 and MobileNetV2). They differ in the number of layers and trainable parameters’ sizes. These networks have very deep networks and can have thousands or even million parameters. The huge number of parameters lets the network learn more difficult patterns, which improves classification accuracy. On the other hand, the huge number of parameters affects the training speed, required memory for saving the network, and computational complexity. MobileNet [48] is an efficient convolutional neural network architecture that reduces the amount of memory used for computing while maintaining a high predictive accuracy. It was developed by Google and trained on the ImageNet dataset. This network is suitable for mobile devices or any devices with low computational power. MobileNetV1 has two layers, which are the depthwise convolution layer for lightweight filtering using a single convolutional filter for each input channel and a 1 × 1 convolution (or pointwise) layer for building new features via computing linear combinations of input channels, while MobileNetV2 consists of two blocks, which are a residual block with a stride of 1 and another block with a stride of 2, that are used for downsizing [49]. Each of these blocks has 3 layers: a 1 × 1 convolution layer with a rectified linear unit (ReLU6), a depthwise convolution, and another 1 × 1 convolution layer without non-linearity.

#### Moving Objects Classification

**Figure 3:**
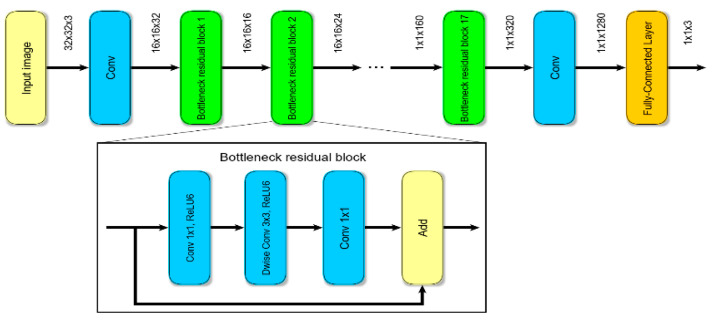
The architecture of the MobileNetv2 network.

One of the most important part of our approach is the classification of found objects. In real-world scenarios, the detected moving objects are drones, birds, airplanes, insects, and moving parts of scenes. Therefore, we decided to use a classifier that divides all found objects into 3 classes: drones, birds, and background. The MobileNetV2 [50] CNN was chosen as the classifier. The choice of the CNN was due to its low value of inference time and high accuracy. According to [51], the highest detection speed was achieved by a detector with the MobileNet [48] backbone network. MobileNetV2 is an improved version of MobileNet that significantly improves its accuracy [50]. The MobileNetV2 network architecture consists of 19 original basic blocks named bottleneck residual blocks (see Figure 3). These blocks followed by a 1 × 1 convolution layer with an average pooling layer. The last layer is a classification layer. We used the modified version of the MobileNetV2 network [52]. The author made the network more suitable for tiny images by changing the stride, padding, and filter size. We changed the classification layer so that the number of classes the network classifies became 3.

## 3. Experiments and Results

### 3.1. Data Preparation

**Figure 4:**
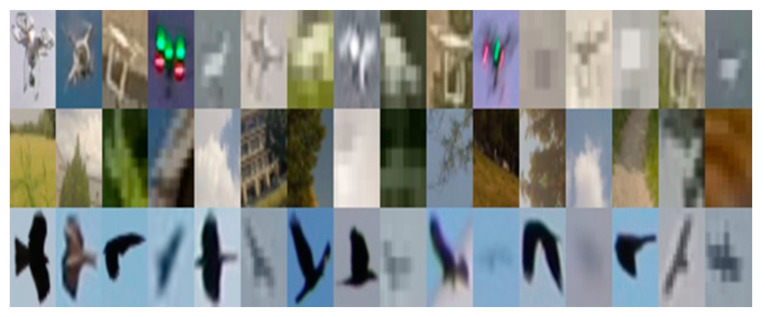
Some of the collected images for training. The first row shows the drones, the second row consists of background images, and the third row is the images of birds.

The amount of data is crucial for CNN training. Insufficient data affect the generalization ability of the network. As a result, the accuracy of the classification is decreased when the network receives new data. As training data in our work, we used the Drone-vs-Bird challenge dataset, which consisted of 11 videos recorded by a static camera. In addition to a drone, birds and other moving objects may appear in the videos. For each video, annotations of drones appearing in the frames are provided. Annotations are presented in the form of coordinates and sizes of the ground truth bounding boxes. Using the videos and annotations to them, we extracted 10,155 images of drones. In order to extract images of birds and background from the videos, we applied the detector of moving objects described in the previous subsection to the entire dataset. Next, we manually labeled all the images of the detected moving objects. As a result, 1921 images of birds and 9348 images of the background were obtained. Since the number of images of birds was low compared to the other two classes, we additionally used 2651 bird images from Wild Birds in a Wind Farm: Image Dataset for Bird Detection [53] Thus, the total number of images in our dataset was 24,075. The input size of the network was 32 × 32 × 3, so we resized all the images to match the input layer of the network. Some examples of the resized images are shown in Figure 4.

### 3.2. Training

We trained the MobileNetV2 CNN from scratch using the dataset described in the previous section. The dataset was divided into a training set and a testing set in a proportion of 80 to 20. To train the network, we used the stochastic gradient descent (SGD) optimization algorithm with a starting learning rate of 0.05, a momentum of 0.9, and a weight decay of 0.001. The training was done on NVIDIA GeForce GT 1030 2 GB GPU with a batch size of 88. We decreased the starting learning rate by a factor of 10 every 50 epochs during the training.

### 3.3. Evaluation Metrics

For the evaluation of any object detection approach, some statistical and machine learning metrics can be used: ROC curves, precision and recall, F-scores, and false positives per image [54]. Generally, first the results of an object detector are compared to the given list of the ground-truth bounding boxes. To answer the question of when a detection can be considered as correct, most studies related to object detection have used the overlap criterion, which was introduced by Everingham et al. [55] for the Pascal VOC challenge. As noted above, the detections are assigned to ground truth objects, and, by calculating the bounding box overlap, they are judged to be true or false positives. In order to be considered as a correct detection according to [55], the overlap ratio between the predicted and ground truth boxes must be exceed 0.5 (50%). The Pascal VOC overlap criterion is defined as the intersection over union (IoU) and computed as follows:

(1) $$IoU=a_{0}=\frac{area\left(B_{p}\cap^{}B_{gt}\right)}{area\left(B_{p}\cup^{\text{​}}B_{gt}\right)}$$

where the IoU is the intersection over union; *a*_0_ is an overlap ratio; *B_p_* and *B_gt_* are predicted and ground truth bounding boxes, respectively; *area*(*Bp* ∩ *Bgt*) means the overlap or intersection of predicted and ground truth bounding boxes; and *area*(*Bp* ∪ *Bgt*) means the area of union of these two bounding boxes. Having matched detections to ground truth we can determine the number of correctly classified objects, which are called true positives (TPs), incorrect detections or false positives (FPs), and ground truth objects that are not missed by the detector or false negatives (FNs). Using the total number of TPs, FPs, and FNs, we could compute a wide range of evaluation metrics.

We evaluated our approach using the Drone-vs-Bird challenge’s [5] metrics. The challenge provided three test videos for evaluation that were named gopro_001, gopro_004, and gopro_006. The first video contained frames with two drones and a moving background. A feature of the second video was a static background and a very small size of the drone. In the third video, in addition to the drone, several birds were present on the frames. The main evaluation metric used in the challenge is the F1-score:

(2) $$F_{1}=2*\frac{Precision*Recall}{Precision+Recall}$$

In order to calculate Precision and Recall, we applied our drone detector on the test videos and counted the total number of TPs, FPs, and FNs.

(3) $$Precision=\frac{TP}{TP+FP}$$

(4) $$Recall=\frac{TP}{TP+FN}$$

Precision and recall are the metrics that can be used to evaluate most information extraction algorithms. Sometimes they might be used on their own, or they might be used as a basis for derived metrics such as F-score and precision–recall curves. Precision is the proportion of predicted positive results that are truly true-positive results for all positively predicted objects, whereas recall is the fraction of all true-positively objects to the total number of positively predicted objects—it shows how many samples of all positive examples were classified correctly. Based on these two metrics, we could calculate F1-score metric, which combines information about precision and recall. A detection was counted as a true positive if the value of the IoU between the detected and the ground truth bounding boxes exceeded 0.5.

### 3.4. Results

**Figure 5:**
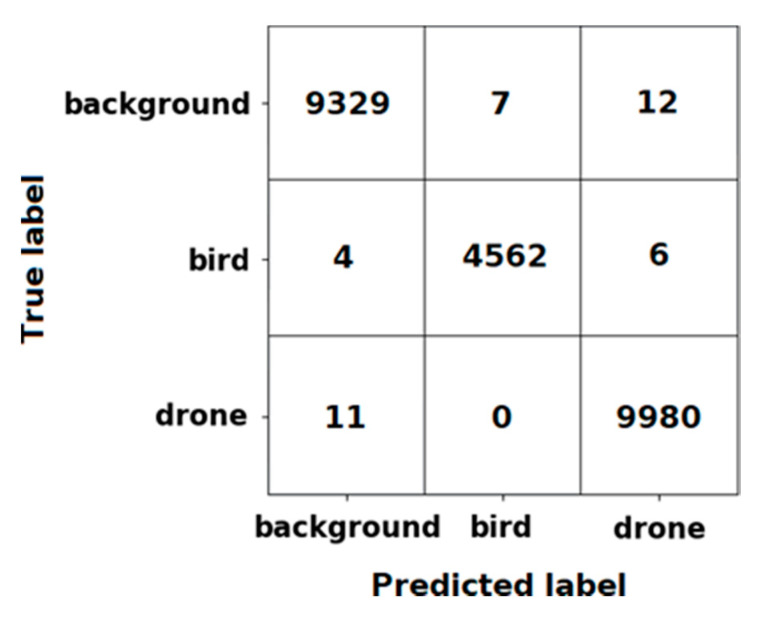
Confusion matrix of the trained convolutional neural network (CNN).

As a result of training, the accuracy of the classifier on the entire dataset was 99.83%. The confusion matrix is shown in Figure 5.

**Figure 6:**
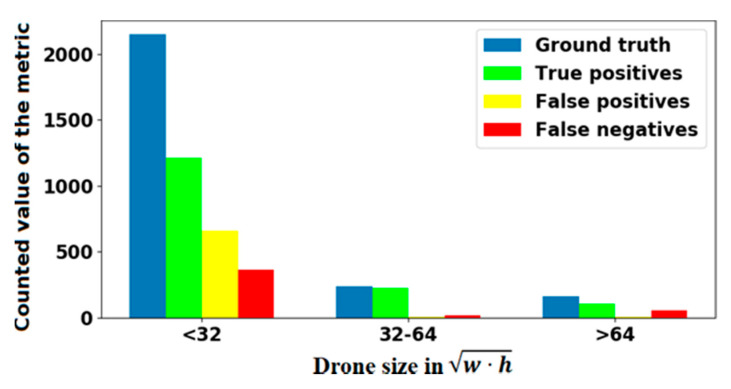
The results of the experiment for various drone sizes.

The experiment results obtained by applying our detector to all test videos are shown in Figure 6. True positives and false positives values were counted for an IoU = 0.5. The results were divided into three ranges, depending on the drone size.

In Figure 6, w and h are the width and height of the ground truth bounding box in pixels, respectively. The value $\sqrt{w*h}\;$ reflects the size of the drone in the image. The lower this value, the farther the drone is from the camera. Based on these data, precision and recall values were calculated. Then, we calculated the F1-score by Equation (1) and added it to the last row of Table 1. The same sequence was individually performed for each video.

**Figure 7:**
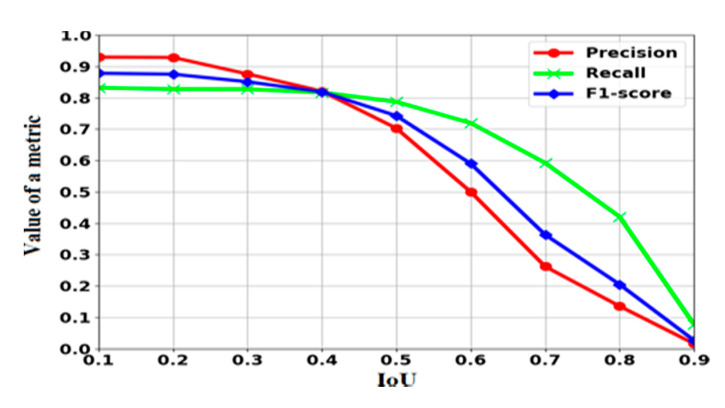
Evaluation metrics values for various IoU values.

For a more detailed analysis of the detector, we conducted experiments for various values of the IoU. The curves were plotted based on the obtained results of recall, precision, and F1-score, as shown in Figure 7.

**Figure 8:**
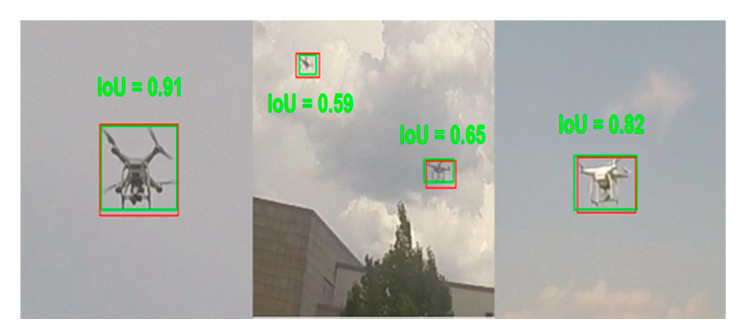
Qualitative results of our detector. Green bounding boxes are ground truth bounding boxes. Red bounding boxes are the results of applying our detector.

Qualitative detection results are depicted in Figure 8.

**Figure 9:**
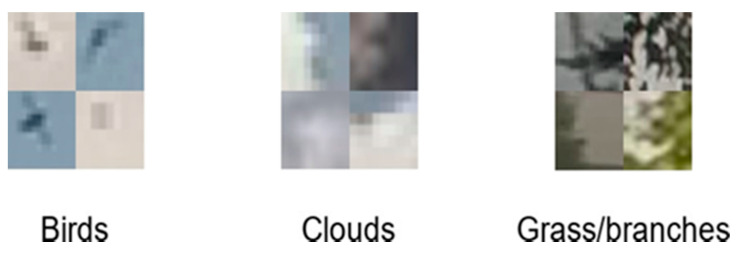
Examples of false detections.

Eighty-five percent of all false positives were caused by inaccurate estimations of the bounding boxes, resulting in a calculated IoU value of less than 0.5. The remaining 15% were classification errors, as a result of which other moving objects were misclassified as drones. Examples of false detections caused by incorrect classification are shown in Figure 9.

**Figure 10:**
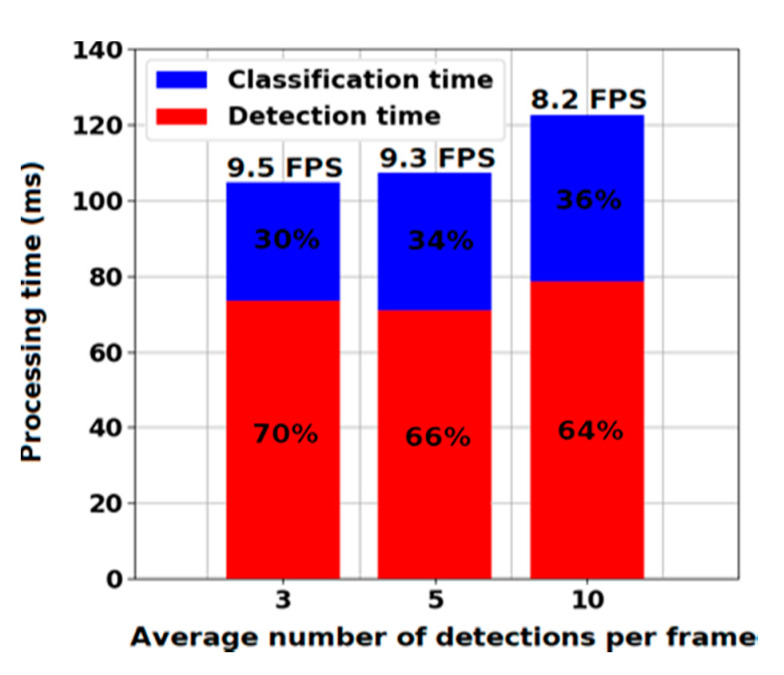
The evaluation results of detection speed.

The images shown in Figure 9 were classified by the detector as drones. In most cases, the objects that were misclassified were birds, clouds, swaying tree branches, and grass. On average, the detector processed nine frames of size 1920 × 1080 per second. A third of the processing time was spent on the classification of moving objects, and the rest was spent on their detection. We noticed that the detection speed depended on the background change rate, which increased as the number of bounding boxes fed into the classifier increased. This dependency is shown in more detail in Figure 10.

## 4. Discussion

**Figure 11:**
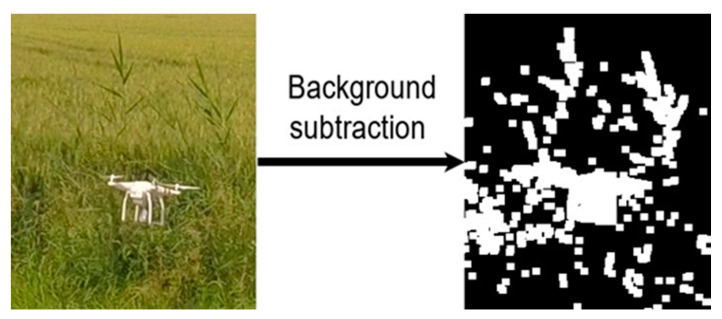
The result of applying background subtraction to the video segment in which a drone was flying near a swaying grass.

Our findings suggest that dividing drone detections task into detecting moving objects and classifying detections can be effective for accurate and fast drone detection. However, the use of motion information for detecting moving objects has several drawbacks. First, as shown in Figure 10, the moving background caused an increase in the number of detected objects, which led to an increase in the classification time and the number of false positives. Secondly, if the drone was flying close to moving objects, then it became impossible to separately distinguish it from other objects, as can be seen in Figure 11.

As a result, the drone was not detected, which led to an increase in the number of false negatives. Along with this, the number of false positive results also increased due to the fact that more images were fed to the classifier. Since the accuracy of the classifier was not equal to 100%, this caused a greater number of classification errors. The usage of the metrics of the Drone-vs-Bird detection challenge [5] allowed us to compare our results with the results of the other teams participating in the challenge. According to the experiment results, the accuracy of our approach was comparable with the approaches proposed in [30] and [14]. Compared to [30], in which only the application of the super resolution was performed at a speed of 0.58 FPS, our detector had a significantly higher detection speed. For IoU = 0.3, our approach performed much worse than [14] but still better than [30]. The comparison results for previous works and our approach are shown in Table 2.

## 5. Conclusions

In this paper, we present a real-time drone detection algorithm, the accuracy of which is comparable to existing algorithms. We provided further evidence that the task of drone detection can be successfully solved by dividing it into the detection and classification stages. The experimental results showed the advantages and disadvantages of this approach. The most important limitation of our detector lies in the fact that its performance is highly dependent on the presence of a moving background. We believe that the accuracy of our detector can be improved by using a larger dataset to train the classifier. For future work, we suggest combining visual information with motion information to detect candidates in the detection stage.

## Figures and Tables

**Table 1:** The results of the evaluation for an intersection over union (IoU) = 0.5.

Video Name	Precision	Recall	F1-Score
gopro_001	0.786	0.817	0.801
gopro_004	0.554	0.910	0.689
gopro_006	0.735	0.691	0.712
Overall	0.701	0.788	0.742

**Table 2:** Comparison results.

Methods	Precision	Recall	F1-Score
[28]	0.103	0.146	0.121
[30]	0.795	0.591	0.678
[14]	0.756	0.713	0.734
[38]	0.524	0.342	0.414
Our approach	0.701	0.788	0.742

## Abbreviations

AI	Artificial Intelligence
CNN	Convolutional Neural Network
COCO	Common Objects in Context
DPM	Deformable Parts Model
DCSCN	Deep CNN with Skip Connection and Network in Network
FPN	Feature Pyramid Network
FPS	Frames per Second
GFD	Generic Fourier Descriptor
GPU	Graphics Processing Unit
HOG	Histogram of Oriented Gradients
IoU	Intersection over Union
KCF	Kernelized Correlation Filter
LBP	Local Binary Pattern
LSTM	Long short-term memory
MPEG4	Moving Picture Experts Group
RAM	Random-access memory
ReLU	Rectified linear unit
RF	Radio Frequency
RGB	Red, Green and Blue
R-CNN	Region-based Convolutional Neural Network
ROC	Receiver operating characteristic
RPN	Region Proposal Network
SAR	Search and Rescue
SIFT	Scale-Invariant Feature Transform
SGD	Stochastic Gradient Descent
SSD	Single Shot Detector
SVM	Support Vector Machine
UAS	Unmanned Aerial System
UAV	Unmanned Aerial Vehicle
USC	University of Southern California
VGG	Visual Geometry Group
VOC	Visual Object Classes
YOLO	You Only Look Once
